# Magnesia (MgO) Production and Characterization, and Its Influence on the Performance of Cementitious Materials: A Review

**DOI:** 10.3390/ma13214752

**Published:** 2020-10-23

**Authors:** Nobre José, Hawreen Ahmed, Bravo Miguel, Evangelista Luís, de Brito Jorge

**Affiliations:** 1 CERIS, DECivil, Instituto Superior Técnico, Universidade de Lisboa, Av. Rovisco Pais, 1049-001 Lisboa, Portugal; jose.nunes.nobre@ist.utl.pt; 2 Department of Highway Engineering Techniques, Erbil Technical Engineering College, Erbil Polytechnic University, Erbil 44008, Kurdistan-Region, Iraq; 3 Scientific Research and Development Center, Nawroz University, Duhok 42001, Kurdistan-Region, Iraq; 4 CERIS, ESTBarreiro, IPS, R. Américo Silva Martinho, 2939-001 Barreiro, Portugal; miguelnbravo@tecnico.ulisboa.pt; 5 CERIS, ISEL, IPL, Rua Conselheiro Emídio Navarro, 1959-007 Lisboa, Portugal; evangelista@dec.isel.ipl.pt

**Keywords:** magnesium oxide, reactivity, chemical properties, physical properties, cementitious materials, mechanical properties, durability, microscopic analysis, literature review

## Abstract

This paper presents a literature review concerning the characteristics of MgO (magnesium oxide or magnesia) and its application in cementitious materials. It starts with the characterization of MgO in terms of production processes, calcination temperatures, reactivity, and physical properties. Relationships between different MgO characteristics are established. Then, the influence of MgO incorporation on the properties of cementitious materials is investigated. The mechanical strength and durability behaviour of cement pastes, mortars and concrete mixes made with MgO are discussed. The studied properties of MgO–cement mixes include compressive strength, flexural strength, tensile strength, modulus of elasticity, water absorption, porosity, carbonation, chloride ion penetration, shrinkage, expansion, and hydration degree. In addition, microscopic analyses of MgO-cement mixes are also assessed. Summarizing the results of different studies, it is concluded that MgO incorporation in cementitious materials generally decreases the mechanical strength and shrinkage, and increases the porosity, expansion, carbonation and chloride ion migration. However, it should be emphasized that the properties of the specific MgO used (mainly the calcination temperature, the reactivity and the surface area) have a significant influence on the characteristics of the cementitious materials produced.

## 1. Introduction

The demand for cement and natural aggregates has been exponentially increasing due to rapid construction development. In fact, in 2014, about 40 billion tonnes of aggregates and 4 billion tonnes of cement were required in the construction sector worldwide [1,2]. Consequently, a significant amount of carbon dioxide (CO_2_) is released to the air during the production stage of these materials. For example, to produce 1 tonne of cement it is necessary to use 125 kW of electricity and to emit about 0.89 tonnes of CO_2_ emissions to the atmosphere [3,4]. One way to overcome this issue is by incorporating sustainable materials, such as fly ash (FA), silica fume, slag, metakaolin, and recycled aggregates in concrete [5,6,7,8,9,10].

Another alternative approach towards sustainable concrete is through MgO incorporation. Cements with high MgO content gained significant popularity in the last decade, mainly due to the growing concern about climate changes, that is, with the intention and the need to mitigate the CO_2_ emissions associated with the production of conventional Portland cements. Some authors believe that it is possible to produce such cements, with a high MgO content, with reduced CO_2_ emissions [11]. Other authors even believe that it is possible to produce cement that has a positive CO_2_ balance, by capturing atmospheric CO_2_ to form magnesium minerals (carbonates and hydroxycarbonates). The carbonation of MgO can be described, in general, as the formation of magnesite from MgO, through the absorption of carbon dioxide [11].

The utilization of MgO can, in some conditions of calcination and reactivity, decrease the thermal shrinkage [12,13], reduce the cost of concrete by decreasing costly cooling measures, and accelerate construction process speed by continuously casting concrete without needing as many cold joints [14]. However, the main motivation for the development and upscaling of MgO-based cements was that of an environmental nature. The lower temperatures required for the production of MgO compared to those required for the conversion of CaCO_3_ into ordinary Portland cement (OPC) and the energy savings associated with that reduced temperature led many to envision MgO-based cements as central to the future of environmentally friendly cement production. Likewise, MgO’s ability to absorb CO_2_ from the atmosphere to form a variety of carbonates and hydroxycarbonates fits well into the discussion of “carbon neutral” cements, which could absorb almost as much CO_2_ during its lifetime as that emitted during their manufacture. These two interconnected aspects have led to a recent rise in interest, both academic and commercial, in the area of MgO-based cements.

Currently, research has been focusing on the mechanical and durability-related properties of cementitious materials with MgO. However, an extensive and critical revision based on MgO characterization and properties of MgO-cementitious materials has not been conducted yet. Therefore, the scope of this review is to present MgO’s characterization in terms of production processes, calcination temperatures, reactivity, chemical and physical properties and microscopic features, and highlight the influence of MgO incorporation in cement pastes, mortars and concrete in terms of mechanical properties (compressive strength, flexural strength, tensile strength, modulus of elasticity), durability behaviour (water absorption, porosity, carbonation, chloride ion penetration, shrinkage), hydration degree, and microstructural analysis. The authors consider that the existence of a review paper that presents the available results up to date in a structured way will be an important support tool to understand whether or not the use of cementitious materials with MgO as replacement of Portland cement is viable in a given application. On the other hand, through the joint analysis of the existing investigations, it is also intended to understand the extension (percentage) of replacement that can be used and which MgO is best suited to the applications evaluated herein.

A very particular strategy was followed in the development of the literature review presented in this paper. First, an initial list of publications was collected, based on several factors: relevance of the title in relation to the topic; cementitious materials type; MgO type; and available data for statistical analysis. For each work collected, an expedient analysis was made in order to establish the relevance of its contents to the research, as well as the tests performed, main outcomes and conclusions. This information was then properly identified and transcribed into a spreadsheet, containing various topics of interest for all publications. As each work was individually evaluated, the relevant data regarding the production of MgO-cementitious material (i.e., cementitious materials type, mix design, curing conditions, etc.) were also collected. Then, an initial table of contents was proposed to serve as a guide for the subsequent investigation. This allowed a comprehensive exploration of the existing information on various factors relating to the use of MgO in the several properties of cementitious materials. As a result, the key points were revealed from the analysis and evaluation data. This allowed drawing several conclusions on the effects of using distinct types of MgO on cementitious materials, and thus enable its use in construction applications.

## 2. Magnesia Production and Its Use in Cementitious Materials

Magnesium is the eighth most abundant element in the Earth’s crust, at ~2.3% by weight, present in a range of rock formations such as dolomite, magnesite and silicate. Magnesium is also the third most abundant element in solution in seawater, with concentrations of ~1300 ppm. The current global production of MgO is 14 million tonnes annually (USGS, 2012), compared with that of OPC of over 2.6 billion tonnes, with current costs of around ~£200/tonne for reactive MgO (calcined), compared to ~£70/tonne for OPC. The cement production process implemented in most industries is known as the dry process and consists mainly of the following steps: grinding and homogenization of raw materials (obtaining raw flour); clinkerization of the raw flour in rotary kilns (clinker production); subsequent clinker cooling; grinding of clinker and addition of gypsum to obtain cement; bagging and shipping of the final product. This process requires high energy consumption and, since it requires temperatures of up to 1400 °C, it emits a large amount of polluting gases. In turn, magnesia (magnesium oxide, MgO) is mainly produced from the calcination of magnesite in a process similar to the production of lime from limestone. A smaller proportion of the world’s MgO production comes from seawater and brine sources, or other sources [15].

### 2.1. Calcination of Magnesite

The most common method used for MgO production is the calcination of magnesite (${\mathrm{MgCO}}_{3}\to \mathrm{MgO}+{\mathrm{CO}}_{2}$) because of the higher energy requirements for production through the wet route. To produce 1 tonne of MgO from fully decomposed pure magnesite, about 1.08 tonnes of CO_2_ can be generated, while OPC production results in 0.85 tonnes of CO_2_ [16,17]. However, the amounts of CO_2_ released and MgO produced are highly dependent on the temperature and CO_2_ pressure used. A kiln with variable temperature is used for magnesite calcination, depending on the required MgO reactivity. In general, four types of MgO are produced [15,16]: light-burned or caustic-calcined MgO (calcined at 700–1000 °C), with the highest reactivity and greatest specific surface area; hard-burned MgO (calcined at 1000–1500 °C), with lower reactivity and specific surface area than those of light-burned MgO; dead-burned MgO or periclase (calcined at 1400–2000 °C), with the lowest specific surface area, making them almost unreactive; fused MgO (calcined at 2800 °C) with the lowest reactivity.

### 2.2. Calcination of Magnesium Hydroxide 

Calcining magnesium hydroxide includes heating a filter cake containing 50–72% magnesium hydroxide solids ($\mathrm{Mg}{\left(\mathrm{OH}\right)}_{2}+\mathrm{Heat}\to \mathrm{MgO}+H_{2}O$). This procedure is similar to that of MgO production in either a brine or seawater process (later discussed in Section 2.3). After placing the filter cake in a kiln, the decomposition reaction starts to take place at 350 °C and it quickly increases above this temperature. During this calcination, several inconsequential processes occur, including filter cake dehydration, dry magnesium hydroxide decomposition, and MgO sintering. The removal of chemically bound water from magnesium hydroxide is a difficult process without raising the temperature above 1000 °C [18].

### 2.3. Seawater and Brine

MgO can be produced from alkaline precipitation of brucite (Mg(OH)_2_) from seawater or Mg rich brine. In the former method, Mg concentration is about 1.4 g/L [19]. The seawater is pre-treated with sulphuric acid to reduce the pH to 4 to remove the carbonates ($\mathrm{Ca}{\left({\mathrm{HCO}}_{3}\right)}_{2}+H_{2}{\mathrm{SO}}_{4}\to {\mathrm{CaSO}}_{4}{\mathrm{CO}}_{2}↑+H_{2}O$). Then, an alkali (lime or sodium hydroxide) is added to raise the pH above the brucite precipitation point (pH 10.5). Sodium hydroxide is used to obtain MgO with low Ca content ($\mathrm{CaO}+H_{2}O+{\mathrm{MgCl}}_{2}\to \mathrm{Mg}{\left(\mathrm{OH}\right)}_{2}↓+{\mathrm{CaCl}}_{2}$). Lime from dolomitic limestone is used to reduce the required additive quantity ($\mathrm{CaO}+\mathrm{MgO}+2H_{2}O+{\mathrm{MgCl}}_{2}\to 2\mathrm{Mg}{\left(\mathrm{OH}\right)}_{2}↓+{\mathrm{CaCl}}_{2}$). After brucite slurry filtration, the filter cake obtained is decomposed at temperatures above 350 °C, requiring higher energy than that of the magnesite calcination method (Section 2.1).

Another MgO production method is by carbonation, involving CO_2_ sequestration through carbonating Mg rich solutions [19,20,21]. For this purpose, natural (seawater or brine) or waste-based solutions (water from oil extraction, rejected brines from a desalinisation process) are used. The reaction between Mg^2+^ and CO_2_ sparged in the solution leads to the precipitation of Mg carbonate. The Mg carbonate type produced depends on the CO_2_ pressure and temperature [20]. For example, the formation of hydrated magnesium carbonates (nesquehonite and hydromagnesite), and magnesite occurs at temperatures of 25 °C, 120 °C and 120 °C, and CO_2_ pressures of 1 bar, 3 bar, and 100 bar, respectively. After that, the obtained Mg carbonate is calcined to formulate MgO.

### 2.4. Extraction of Magnesia from Mg-Bearing Minerals

This method involves geological CO_2_ sequestration, where rocks containing chemical groups capable of carbonation are decomposed to suitable precursors to react with CO_2_ [22,23,24]. The magnesium silicates decomposition can be facilitated by various methods. The first one is through Mg silicate acid digestion (${\mathrm{Mg}}_{3}{\mathrm{Si}}_{2}O_{5}{\left(\mathrm{OH}\right)}_{4}\left(s\right)+6\mathrm{HCl}\left(\mathrm{aq}\right)\to 3{\mathrm{MgCl}}_{2}\left(\mathrm{aq}\right)+2{\mathrm{SiO}}_{2}\left(s\right)+5H_{2}O\left(l\right)$), followed by brucite precipitation (${\mathrm{MgCl}}_{2}+2\mathrm{NaOH}\to \mathrm{Mg}{\left(\mathrm{OH}\right)}_{2}+2\mathrm{NaCl}$), and its calcination ($\mathrm{Mg}{\left(\mathrm{OH}\right)}_{2}\to \mathrm{MgO}+H_{2}O$) [22]. The second method is through Mg silicate carbonation with subsequent Mg carbonate calcination (${\left(\mathrm{Mg}.\mathrm{Ca}\right)}_{x}{\mathrm{Si}}_{y}O_{x+2y}+{\mathrm{xCO}}_{2}\to x\left(\mathrm{Mg}.\mathrm{Ca}\right){\mathrm{CO}}_{3}+{\mathrm{ySiO}}_{2}$) [24]. The Mg carbonate type from this process depends on the CO_2_ pressure and temperature. For example, formulation of magnesite occurs at 155 °C and 126 bar, and hydrated magnesium carbonates at lower temperature and pressure [24]. After that, the carbonates would be calcined to produce MgO, CO_2_ and possibly H_2_O.

### 2.5. MgO in Cementitious Materials

In general, two main methods are used to add MgO in cementitious materials. One is by increasing the periclase (magnesium oxide mineral) content in cement clinker to produce high magnesia cement. This method has been used in dam concrete for about 40 years in China [25]. The second method is by preparing MgO from magnesite (MgCO_3_) calcination and then incorporating the material in concrete as an expansive additive [25]. When using the second method, it is important to homogenously disperse MgO in concrete by using an adequate mixing process to avoid heterogeneous expansion that could lead to concrete destruction.

The addition of MgO to conventional Portland cements results in the formation of Mg(OH)_2_ ($\mathrm{MgO}+H_{2}O\to \mathrm{Mg}{\left(\mathrm{OH}\right)}_{2})$ and its subsequent carbonation ($\mathrm{Mg}{\left(\mathrm{OH}\right)}_{2}+{\mathrm{CO}}_{2}+2H_{2}O\to {\mathrm{MgCO}}_{3}.3H_{2}O)$, giving rise to hydrated magnesium carbonates. This type of cement was designed to replace Portland cement in large quantities, thereby deriving environmental benefits with respect to CO_2_ emissions. However, due to the long-term dimensional instability seen in concrete with cements with high MgO content, existing standards strictly limit the MgO content that can be used in Portland cements [11].

## 3. Magnesia Characterization

### 3.1. Physical Properties

MgO is commonly used as a raw material for Portland cement. This MgO is obtained by calcination at 1400–2000 °C of MgCO_3_, when clinker is produced. These high temperatures allow a large crystalline structure to be formed, with reduced reactivity and hydration rate. This behaviour causes a slow expansion, leading to extensive cracking of the cement paste [26]. Due to the referred behaviour, the standard EN 197 indicates that the MgO content in the cement should not exceed 5% (of cement mass). However, by varying the calcination temperature, MgO with completely different physical-chemical properties can be obtained.

The main physical properties of MgO are summarized in Table 1. MgO thermal conductivity has been determined by different studies, covering polycrystalline sintered MgO at lower temperatures [27] and sintered MgO at higher temperatures [28]. Table 1 shows values between 0.03 and 0.10 cal s^−1^ cm^−2^ °C^−1^ cm.

It was also found that MgO’s electrical resistance is very high, which makes it an excellent high-temperature electrical insulator. The relationship between specific resistance (*ρ*) of magnesia and temperature (*T*) can be expressed by $\left(\rho =Ae^{B/T}\right)$, where *A* and *B* are specific constants. Table 1 displays the specific resistivity for sintered and high-purity MgO.

The thermal expansion of periclase is the greatest of all pure refractory oxides and approaches the expansion of metals. Expansion measurements have been carried out on single periclase crystals and high-purity sintered MgO (Table 1). Values for the specific heat capacity from ambient to higher temperatures are also shown in Table 1.

Regarding the structural properties, MgO has compressive strength of 0.83–1.44 GPa, tensile strength of 96 MPa, elastic modulus of 210–317 GPa, and flexural strength of 90 MPa, according to general references, as reported by Shand [16].

### 3.2. Reactivity

MgO reacts with water and diluted acids, and its reactivity (rate and degree of reaction) depends considerably on the physical properties and purity of the material [33]. MgO reactivity increases by reducing its particle size and, consequently, increasing its specific surface area [33]. MgO surface area and particle size are both controlled by the production conditions (raw material type and purity, calcination temperature, and residence time during calcination). Similar to other types of metal oxide, MgO has various surface defects, significantly influencing their reactivity [34]. The usual one-dimensional surface defects are in the step-shape and may have defect points [16]. In general, two types of MgO defects have been investigated: oxygen vacancy and Mg vacancy [16]. MgO reactivity is also influenced by the chemical nature of the precursor from which MgO is produced [35]. The arbitrary industrial classification of MgO reactivity serves for the appropriate grade selection depending on the application from hard-burned (slow hydration) to reactive (fast hydration) [16].

MgO reactivity is assessed according to the neutralisation rate of weak acid solutions, including citric acid [36] and acetic acid [16]. In the former testing method, citric acid is dissolved in distilled water, followed by adding Bromothymol blue (pH indicator) and MgO. The time taken for the solution to change colour is measured. For the latter testing method, MgO is added into distilled water, followed by adding phenolphthalein indicator and acetic acid with continuous mixing. The time is measured until development of the red phenolphthalein colour.

**Figure 1:**
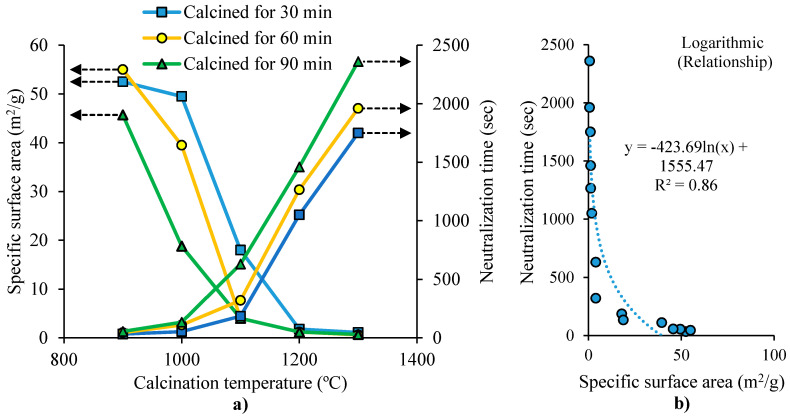
(**a**) Effect of calcination temperature and residence time on the surface area and neutralization time of MgO. (**b**) Relationship between the surface area and the neutralization time of MgO (adapted from Mo et al. [37]).

For MgO production, MgCO_3_ undergoes calcination in a furnace at a given temperature (calcination temperature), and a residence time (calcination time). The activity of the resulting MgO is evaluated by measuring the time needed to fully neutralize an acidic solution (neutralization time). MgO with higher reactivity tends to need shorter neutralization time [37]. The surface area and neutralization time of MgO are influenced by the time and temperature of calcination, as shown by Mo et al. [37]. In their study, magnesite was crushed into fine particles (<80 μm) and calcined in a furnace at a given temperature and calcination time. Figure 1a shows that the specific surface area of MgO decreases by increasing the temperature and residence time (calcination time). This was attributed to the particle growth of MgO due to the continued sintering, leading to total pore volume reduction and pore size enlargement. Figure 1a also shows that the neutralization time of MgO increases by increasing the calcination temperature and the residence time. The residence time has higher effect on the neutralization time, at higher temperature. Figure 1b shows the inverse relationship between MgO’s neutralization time and surface area. The neutralization time decreased considerably when the surface area was smaller than 1.3 m^2^/g. This decrement became less apparent for specific surface areas bigger than 20 m^2^/g. This was attributed to the influence of the surface area and hydration activity of MgO particles (related to its crystal size) on the MgO activity. In the study of Wogelius et al. [38], it was reported that MgO hydration activity was related to its surface structure, meaning that low-defect surface MgO had lower activity. At an early stage of sintering, more defects exist in MgO, leading to higher grain activity. MgO crystal lattice’s atoms array gradually become regular, contributing to fewer defects and lower hydration activity.

Mo et al. [37] reported that the density of MgO and MgO grain size increased with the increase of calcination temperature and residence time, while MgO lattice distortion decreased (Table 2). The study also investigated the hydration degree of MgO for samples with 60 min of residence time, at 2, 7, 15 and 30 days, using microscopic analysis (Table 2). The results showed that the most rapid hydration occurred at 900 °C, where about 97% and 100% of MgO has been hydrated at 2 and 7 days, respectively. For the sample at 1100 °C, MgO hydration degree was 4.5% and 9.9% at 2 days and 7 days, respectively. The sample at 1300 °C had the slowest hydration. In conclusion, MgO grain hydration activity has higher influence than its surface area on MgO hydration activity.

## 4. Characterization of Cementitious Materials with Additional MgO

Several experimental campaigns conducted over the years have proven that the performance of concrete produced with cements with reactive MgO can be quite interesting. So, in the next sub-chapters, the results obtained by several investigations on the properties of such concrete mixes are analysed.

### 4.1. Mechanical Properties

The mechanical properties of cement pastes, mortars and concrete mixes produced with different types and amounts of MgO, studied by different researchers, are summarized in Table 3. In general, incorporating MgO leads to strength decrease of cement pastes, mortars and concrete. In fact, this behaviour was observed in compressive strength [39,40,41,42,43,44,45,46], flexural strength [42,43,46,47], and tensile strength [48]. The incorporation of higher amounts of MgO caused further strength decrements [39,43,46,47,49].

Liu et al. [39] studied the influence of calcined MgO addition in cement pastes. Mixes were produced with 0%, 2% and 3% of MgO and water to binder ratio (w/b) of 0.265. The compressive strength of the cement pastes was tested at 3, 7 and 28 days. There was a compressive strength reduction with increased MgO content and age of samples, relative to a reference paste without additional MgO (ordinary Portland cement mix).

Abdalqader and Al-Tabbaa [40] studied the compressive strength of cement pastes made with OPC (30%, 75%), FA (15–25%), slag (45–60%), and MgO (5%, 10%) with 170 s reactivity according to the acetic acid test, at 3, 7, 28 and 56 days. The average compressive strength of mixes containing OPC (75%) and FA (25%) was higher than those containing FA, slag, and MgO, at all ages. This was attributed to the smaller amount of OPC in the latter binders to activate FA and slag. It was also found that the use of 10% MgO, 60% slag and 30% Portland cement gave rise to cement pastes with strengths similar to those with 75% Portland cement and 25% fly ash. Therefore, it can be concluded that the combined use of MgO and slag causes the occurrence of chemical reactions that favour the strength of these cementitious materials.

Mo et al. [42] studied the effect of MgO with 50 s reactivity according to the citric acid test on the flexural and compressive strength of mortars. Mixes were produced with 5% and 8% of MgO as a replacement for cement clinker during the inter-grinding process. The addition of MgO induced reductions of the flexural and compressive strength at all ages. At 90 days, the compressive strength in mortars with 5% and 8% of MgO decreased by 13.7% and 19.7%, respectively, in comparison with that of conventional mortars. The authors attributed the reduction to the less frequent C–S–H formation due to OPC reduction in MgO mixes.

In summary, the results available to date in the literature on the mechanical behaviour of cementitious materials with reactive MgO allow the following conclusions:

—Compressive strength generally decreases with the introduction of MgO regardless of the replacement rate and reactivity of MgO. This reduction is of about 10% and 30%, when using 5% and 20% of MgO, respectively;

—Flexural strength also decreases with the use of reactive MgO in detriment of Portland cement. This reduction is similar to that found for compressive strength;

—The modulus of elasticity slightly decreases with the use of reactive MgO instead of Portland cement. This reduction equals 10% when 20% of MgO is used. This decrease is most likely due to the higher quantity of water required to obtain similar workability levels. Some investigations even point to the maintenance of this property with the use of MgO;

—The generalized decrease in mechanical properties is due to less frequent C–S–H formation due to OPC reduction in MgO mixes;

—Even though this decrease is generally observed at any age, it appears that the use of MgO in cementitious materials makes them approach their final strength earlier, especially when using highly reactive MgO;

—The joint introduction of MgO and slag causes the occurrence of chemical reactions that favour the strength of these cementitious materials;

—The carbonated curing conditions allow cementitious materials with MgO to reach a higher increase of their mechanical strength than cementitious materials without MgO. This was attributed to the higher porosity in the first mixes, leaving more space for hydrated magnesium carbonate formulation and strength development;

—The incorporation of different HA types (magnesium acetate, magnesium chloride, hydrochloric acid) in MgO concrete enabled higher strength development increments, when compared with the same incorporation in conventional concrete mixes.

### 4.2. Durability Behaviour

The durability behaviour of cementitious materials with MgO, studied by different researchers, is summarized in Table 4. In general, the incorporation of MgO leads to lower water absorption [45,47,52], higher carbonation [41,48], higher chloride ion migration coefficient [41], higher initial expansion [37,39,42,43,46,47,57,58], and lower shrinkage [42,43,53].

Moradpour et al. [47] studied the water permeability of mortars produced with nano-MgO, at 28 days. The results showed that the permeability decreased in mortars containing nano-MgO particles. The researchers defined 1% of MgO as an optimal amount to obtain maximum improvements of the mechanical strength and water absorption of mortars. Dung and Unluer [51,52] also studied the water absorption of concrete mixes produced with MgO and found a decrement when compared to that of conventional mixes.

Mo and Panesar [56] studied the porosity of cement pastes produced with 10%, 20% and 40% reactive MgO (calcined at 800 °C), under carbonation and non-carbonation conditions. The researchers found that incorporating 20% of MgO could lead to 32% pore volume decrease at 28 days, when compared to that of reference cement paste, under accelerated carbonation. By contrast, a 6–10% pore volume increase was found in pastes with 10–40% MgO, under non-carbonation condition.

Liu et al. [39] investigated the expansion behaviour of cement pastes produced with 2%, 3%, 4%, 5% and 6% of MgO. The length variation was measured in small prisms (10 × 10 × 40 mm) up to 210 days, at two different temperatures (20 °C and 50 °C) in water. The expansion increment rapidly took place at early ages (1–30 days), it slowed down between 30 to 90 days and became even after 90 days. Cement pastes presented higher expansion values with the increment of MgO content and curing temperature.

The results available to date in the literature on the durability-related behaviour of cementitious materials with reactive MgO, prompt the following conclusions:

—Porosity generally increases with the incorporation of MgO regardless of the replacement ratio and of its reactivity. This increase will be higher than 10% when using more than 5% MgO. However, the results in the literature are quite variable, with some studies showing improvements in this property with the use of MgO at ratios lower than 5%;

—Carbonated curing conditions allow cementitious materials with MgO to decrease their porosity to a higher extent than that found for cementitious materials without MgO. This was attributed to the higher initial porosity in the first mixes, leaving more space for hydrated magnesium carbonate formulation;

—Carbonation depth increases with the use of reactive MgO to the detriment of Portland cement. This increase can reach up to 400% for ratios of 20% MgO;

—The MgO may have little effect on the freeze/thaw resistance of concrete. The authors justify this through MIP analysis that indicate that pores larger than 10 µm within the MgO concrete do not present any changes;

—The expansion of cementitious materials is widely controlled by the MgO content, neutralization time, curing temperature, and MgO reactivity level. Overall, higher MgO content leads to higher initial values of expansion. MgO with higher reactivity led to higher expansion;

—As for shrinkage, the MgO incorporation effect in cementitious materials is dependent on the MgO reactivity [38,41] and MgO content [38,40]. However, the shrinkage is always lower with the incorporation of MgO. The use of 15% of MgO as cement replacement led to remarkable decreases in shrinkage, which may be of only about 10% of the 91-day shrinkage of OPC concrete.

### 4.3. Hydration Degree

**Figure 2:**
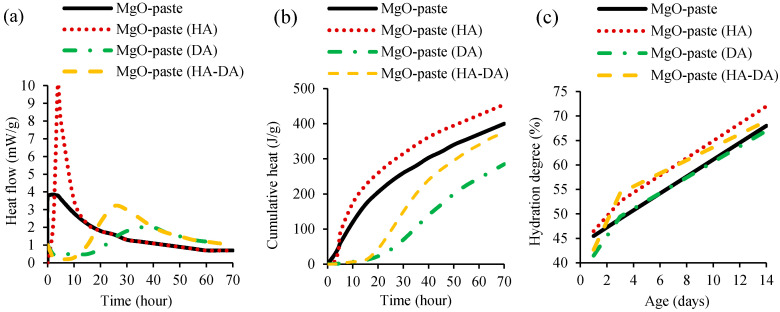
Isothermal calorimetry of MgO-cement pastes showing (**a**) heat flow and (**b**) cumulative heat. (**c**) Thermogravimetric analysis (hydration degree) of MgO-cement pastes (adopted from [51]).

The hydration of cementitious materials produced with additional MgO was studied by Dung and Unluer [52] and Mo et al. [42] through isothermal calorimetry and thermogravimetric analysis. In the study of Dung and Unluer [51], MgO-cement pastes were produced with and without HCI (hydration agent, HA) and NaHMP (dispersion agent, DA). The heat flow (Figure 2a) and cumulative heat (Figure 2b) of cement pastes were analysed through isothermal calorimetry at 30 °C for 72 h. Thermogravimetric analysis was assessed to evaluate MgO hydration degree with and without DA and HA, under up to 600 °C at 1, 3 and 14 days (Figure 2c).

The isothermal calorimetry results showed that MgO dissolution and brucite formation occurred after the first few hours of mixing MgO-cement pastes with and without HA. This first peak of MgO-cement paste with HA was significantly higher than that of the mix without HA. The addition of DA in mixes with and without HA slowed down this action to 10 to 15 h, followed by a lower peak of hydration and MgO dissolution. After 24 h and 38 h, a wide brucite formation peak was observed in mixes containing both DA and HA and only DA, respectively. In conclusion, the incorporation of HA increased the MgO hydration, while the opposite effect was observed with the addition of DA, associated with the deflocculating effect of the latter. The thermogravimetric analysis (Figure 2c) also showed hydration increase with the addition of HA in mixes with or without DA, at 3 and 14 days, when compared to that of reference mix without HA and DA. The same authors [52] also investigated the influence of using 0.05% and 1% of different HA types, namely magnesium chloride (MgCl_2_), magnesium acetate ((CH_3_COO)_2_Mg), and hydrochloric acid (HCl). MgO-cement pastes containing HA obtained higher values in terms of heat flow, cumulative heat and hydration degree, when compared to mixes without HA. The incorporation of HAs enhanced the dissolution of reactive MgO and the precipitation of brucite, which, in turn, increased the rate and degree of reactive MgO hydration. Increasing the amount of HA led to increase of hydration. The isothermal calorimetry results showed that the maximum increases was achieved with the addition of MgCl_2_, followed by (CH_3_COO)_2_Mg, and HCl. In the thermogravimetric analysis, the sequence was (CH_3_COO)_2_Mg, MgCl_2_, and HCl.

Mo et al. [42] made an isothermal calorimetry analysis of cement pastes made with 8% MgO, with or without 20–0% slag and 20–35% FA, during the first 72 h after mixing. In the first 7 h, no obvious differences were observed between the heat flow of the reference mix without MgO and MgO mixes. This was attributed to the accelerated formation of C-S-H. However, the addition of MgO led to decrease of maximum heat flow peak between 7–12 h and increase of heat flow between 12–48 h. Compared to the reference cement paste and that containing only MgO, mixes with FA, slag, and MgO presented longer periods of acceleration and induction. In fact, the MgO–cement pastes with 40% slag and 20% FA and the one with 20% slag and 35% FA reached their maximum heat flow 2 h after reference mix. This was attributed to small cement hydration retardation caused by slag and FA.

### 4.4. Microscopic Analysis

The analysed observations based on scanning electron microscopy (SEM) of cementitious materials made with additional MgO, collected from different studies, are summarized in Table 5. Each study presented different aspects and phenomena, including the ability of MgO to create cracks in cementitious materials [37] and densify its microstructure [47,49].

Mo et al. [37] assessed the morphology of cement pastes produced with MgO having different neutralization times (46, 325, and 1966 s), water-cured for 270 days at 40 °C. Due to the influence of alkali on MgO hydration, the hydration products in Mg(OH)_2_ were more irregular and smaller, when compared to reference paste without additional MgO. On the other hand, cracks were observed at the sintered MgO particle interface, associated with its expansion at the particle boundary. The same researchers [43] produced cement pastes with incorporation of two MgO types (reactivity values: 50 and 400 s), FA and slag, and cured them in 38 °C water. In mixes without FA and slag, the MgO particles were surrounded by hydration products of cement. Sheets with similar features of brucite were observed, with some empty inner pores. In mixes with FA and slag, rims of pozzolanic reaction were mainly formulated around coarser slag particles. The finer slag particles appeared to be fully hydrated.

Moradpour et al. [47] observed that mortars produced with 1%, 3% and 5% nano-MgO had denser microstructure than that of the reference mix. The authors attributed this fact to the expansion and filler effects of MgO. The backscattered electron mode of SEM showed modified C–S–H in mixes with MgO.

## 5. Conclusions

From the review of published literature focused on the mechanical strength and durability behaviour of cement pastes, mortars and concrete mixes produced with additional MgO, the following main conclusions were drawn:

- The compressive strength, flexural strength and tensile strength of cementitious materials decreased with the incorporation and increase in the MgO content, regardless of the material being added directly to the mix or to the cement clinker. This was mainly attributed to the porosity increment and lower hydration of the MgO mixes, when compared to conventional mixes without MgO. The reactivity of MgO showed insignificant influence on the strength of cementitious materials;
- The incorporation of MgO could lead to porosity decrease, when compared to that of conventional reference mixes, under accelerated carbonation. By contrast, porosity increased with the addition of MgO under ambient carbonation condition;
- The carbonation of concrete mixes produced with MgO tends to be higher than that of conventional concrete mixes. This trend becomes more evident with higher MgO incorporation levels;
- The chloride ion migration coefficient increased by incorporating MgO in mixes water-cured for 28 days and decreased in those water-cured for 360 days. This decrease was attributed to the reduced porosity with the addition of MgO in mixes cured for 360 days;
- The initial expansion of concrete mixes increased by increasing the MgO content;
- The shrinkage of cementitious materials decreased with the incorporation of MgO due to the compensation of the shrinkage by MgO hydration, during 1–5 days. The shrinkage of cementitious materials fell significantly by increasing the reactivity of MgO;
- The hydration degree of cementitious material mixes was not changed by the addition of MgO, during the first 7 h of mix production. This was attributed to the accelerated formation of C–S–H. However, the addition of MgO led to a decrease of maximum heat flow peak between 7–12 h and increase of heat flow between 12–48 h. The incorporation of hydration agent increased the MgO hydration, while the opposite effect was observed with the addition of dispersion agent, associated with the deflocculating effect of the latter;
- Microscopic analysis showed that cementitious materials produced with MgO may have had denser microstructure when compared to that of conventional reference mixes. This was attributed to MgO hydration products filling the pores and to the expansion effect of MgO.

In the future, it would be interesting to prepare a systematic literature review by subjecting the existing data to a statistical analysis. Based on the type and content of the reactive MgO, the existing results allow the development of models for predicting different properties.

## Figures and Tables

**Table 1:** Physical properties of magnesia.

Temperature (°C)	Thermal Conductivity of Polycrystalline Sintered Magnesia (cal s^−1^cm^−2^°C^−1^cm) [27]	Thermal Conductivity of Sintered Magnesia at High Temperatures (cal s^−1^cm^−2^°C^−1^cm) [28]	Average Thermal Expansion Coefficient (L) of Single-Crystal Periclase (×10^−6^/°C) [29]	Thermal Expansion Coefficient (L) of High-Purity Sintered Magnesia (×10^−6^/°C) [30]	Specific Heat Capacity (J·K^−1^ mol^−1^) [16]	Specific Electrical Resistance of Sintered Magnesia (×10^3^ Ω) [16,31]	Specific Electric Resistance of High-Purity Magnesia (Ω) [32]
0	0.1	-	-	-	-	-	-
25	-	-	-	-	37.1	-	-
50	-	-	6.7	-	-	-	-
100	0.083	-	9.1	-	-	-	-
300	0.067	-	11.6	12.0	-	-	-
327	-	-	-	-	47.4	-	-
500	0.031	-	-	12.6	-	-	-
600	0.026	-	13.0	-	-	-	-
700	-	-	13.2	13.2	-	-	-
727	-	-	-	-	51.2	-	-
800	-	-	13.5	-	-	-	-
900	-	-	13.7	13.7	-	-	9 × 10^7^
950	-	-	-	-	-	120	-
1000	-	0.1	13.8	-	-	95	-
1100	-	-	-	14.7	-	-	-
1200	-	0.083	-	-	-	-	-
1300	-	-	-	14.5	-	9	2.5 × 10^5^
1400	-	0.067	-	-	-	-	-
1500	-	-	-	15.0	-	1.5	-
1527	-	-	-	-	54.9	-	-
1600	-	0.031	-	-	-	-	-
1700	-	0.026	-	15.6	-	-	5.5 × 10^3^
1800	-	-	-	16.0	-	-	-
2100	-	-	-	-	-	-	4.4 × 10^2^
2527	-	-	-	-	58.5	-	-
3327	-	-	-	-	61.2	-	-

**Table 2:** Influence of calcination temperature (calc. temp.) and residence time (res. time) on microstructure and hydration degree of MgO [37].

Sample	Crystal Grain Size (nm)	Lattice Distortion (%)	Specific Surface Area (m^2^/g)	Pore Volume (cm^3^/g)	Average Pore Width (nm)	Density (g/cm^3^)	Hydration Degree			
							2d	7d	15d	30d
Calc. Temp. 900-Res. Time 30	21.2	0.1648	52.7	0.193	14.62	2.97	-	-	-	-
Calc. Temp. 900-Res. Time 60	29.3	0.0560	55.2	0.223	16.10	3.02	97.1	100	100	100
Calc. Temp. 900-Res. Time 90	36.8	0.0350	45.9	0.179	15.57	3.12	-	-	-	-
Calc. Temp. 1000-Res. Time 30	30.4	0.0208	49.8	0.235	18.84	3.13	-	-	-	-
Calc. Temp. 1000-Res. Time 60	43.2	0.0149	39.8	0.235	23.61	3.26	-	-	-	-
Calc. Temp. 1000-Res. Time 90	66.3	0.0092	18.9	0.103	25.12	3.32	-	-	-	-
Calc. Temp. 1100-Res. Time 60	>100	-	4.2	0.024	22.46	3.43	4.5	9.9	20.9	82.6
Calc. Temp. 1300-Res. Time 60	-	-	-	-	-	-	-	1.0	1.9	7.9

**Table 3:** Mechanical properties of cementitious materials with MgO.

Test	Reference	Mix	Binder (%)					w/b	MgO		Age (days)	Results
			OPC	FA	S	MS	MK		(%)	Properties		
Compressive strength	Liu et al. [39]	P	100	-	-	-	-	0.265	1–5	-	3–28	The strength of pastes with MgO reduced up to 15% with the increase of age and of MgO content, compared to the reference pastes (ordinary Portland cement).
	Jin et al. [49]	P	-	-	100	-	-	0.31	2.5–7.5	Reactivity 10–220 s	1–90	The strength of alkali-activated slag cement increased with increasing the amount of high reactivity MgO. Unclear trend was observed with increasing the amount of medium reactivity MgO, at different ages. However, among different amounts of medium reactivity MgO, mixes containing 2.5% obtained maximum increases at 7–90 days, when compared to the strength of control mix.
-	Abdalqader and Al-Tabbaa [40]	P	25–75	15–25	45–60	-	-	0.31	5–10	Reactivity 170 s	3–56	The average strength of mixes containing only FA was higher than those containing FA, slag, and MgO, at all ages. Thus, maximum strength was registered in mixes with 25% FA and 75% OPC, followed by those containing 30% OPC, 60% slag, and 10% MgO.
-	Mo et al. [41]	P	10–60	20–40	-	-	-	0.4	20–60	Calcination 800 °C	28	Cement pastes were exposed to different CO_2_ periods and CO_2_ pressures (0.55 MPa for 3 and 15 h, and 0.10 MPa for 1 and 14 days). In mixes without CO_2_ exposure, the strength decreased with increasing the amount of FA and MgO. Exposing cement pastes to 0.55 MPa CO_2_ for 3 h led to 7–72% strength increase. In that case, higher replacement of cement caused higher strength increments with CO_2_ exposure. The strength of mixes exposed to 0.55 MPa CO_2_ for 3 h was higher than that of mixes under 0.10 MPa for 1 day. In general, the strength of cement pastes increased with increasing CO_2_ pressure or elongating the exposure time.
-	Mo et al. [42]	M	100	-	-	-	-	0.5	5–8	Reactivity 50 s	3–90	Reduction of strength occurred with increasing the amount of MgO at all ages, when compared to that of reference mortars. This was attributed to the lower C-S-H formation due to cement weight reduction and replacing it by MgO.
-	-	-	25–100	20–35	20–40	-	-	0.5	5–8	Reactivity 50 s	3–90	At 3 days, the incorporation of slag and FA significantly decreased the strength of reference and MgO mixes. This difference decreased at 28 and 90 days. Comparing the strength of the same mixes at 90 days to 3 days, the strength development of 8% MgO mixes with FA and slag (157–313%) were higher than that of 8% MgO-mortars without FA and slag (47%). This was attributed to pozzolanic or hydraulic reaction of FA and slag. Higher FA amount decreased the strength while higher slag increased it.
-	Mo et al. [43]	M	100	-	-	-	-	0.5	8	Reactivity 50–400 s	3–90	The incorporation of four MgO types (reactivity values: 50, 100, 200, 400) caused slight strength reduction of cement mortar, particularly at late age, when compared to that of conventional reference mortar. The reactivity of MgO showed insignificant influence on the strength of cement mortars.
-	-	-	40–100	20	40	-	-	0.5	8	Reactivity 50–400 s	3–90	The strength of reference and MgO-mortars significantly decreased with the addition of FA and slag at 3 days. This difference highly decreased at 28 days. At 90 days, the strength of MgO-mortars with FA and slag was slightly higher than that of reference and MgO-mortars without FA and slag.
-	Moradpour et al. [47]	M	100	-	-	-	-	0.5	1–5	-	7–90	The strength increased in mortars with nano-MgO, regardless of curing age and MgO content, when compared to that of conventional reference mortars. In fact, MgO mortars had 1.1–2.0 times higher strength. This trend was more obvious with lower MgO content.
-	Wei et al. [50]	M	-	-	-	10–40	-	0.5	60–90	-	3–28	Mortars were produced without OPC and with different amounts of microsilica and MgO. Maximum strength was obtained in mixes containing 30% microsilica and 70% MgO, at 28 days. This confirmed that full replacement of cement by microsilica and MgO can lead to production of mixes with the same strength as conventional mortars.
-	Dung and Unluer [51]	C	100	-	-	-	-	0.5–0.6	-	-	3–28	The incorporation of HCI (hydration agent-HA) and NaHMP (dispersion agent, DA) agents in concrete mixes containing MgO-based cement increased compressive strength up to 50% when compared to that of control mix. This trend was more pronounced in mixes containing only DA, followed by those with HA and DA, and only HA.
-	Dung and Unluer [52]	C	100					0.6	-	-	3–28	The incorporation of different HA types (magnesium acetate, magnesium chloride, hydrochloric acid) and HA concentrations (0.05–0.1 M) into MgO concrete enabled strength development increments up to 107% and 53%, when compared to that of the corresponding MgO-concrete mixes (without HA) and conventional concrete mixes, respectively. This trend was more obvious under accelerated curing conditions than under ambient conditions.
-	Pu and Unluer [48]	C	100	-	-	-	-	0.38–0.80	5–10	-	1–28	The strength of concrete blocks made with 90–95% aggregates and 5–10% MgO or OPC was investigated under carbonation. Maximum strength was obtained in concrete with 10% MgO, followed by those with both MgO (5%) and OPC (5%). The strength of concrete containing 10% MgO was 83% higher than that of concrete with 10% OPC after 7 days.
-	Choi et al. [44]	C	80	20	-	-	-	0.48–0.65	5	Calcination 1000 ºC	7–540	The addition of 5% MgO into concrete mixes produced with 20% FA and different w/b (0.48, 0.65) led to strength decrease (4–13%) at 7 and 28 days. This was attributed to the slow MgO hydration up to 28 days tested in MgO-mortars with X-ray analysis. At 56–540 days, the strength of MgO-concrete mixes became similar to that of the reference mixes.
-	Mavroulidou et al. [45]	C	40–80	20–25	-	-	15-25	0.55	5–10	Reactivity 976 s	28	Strength of concrete mixes produced with different amounts of FA and metakaolin slightly decreased with increasing the amount of MgO. This was attributed to MgO’s contribution to porosity increase and production of low strength magnesium silicate hydrates, and lack of reaction between FA and brucite to formulate M-S-H gel, and between cement and MgO to formulate more products.
-	Unluer and Al-Tabbaa [53]	C	-	5	-	-	-	0.67–1.44	10	-	7	Cement was produced with 5–10% of MgO and 0–5% of heavy or light hydrated magnesium carbonates. Concrete blocks were produced with 10% cement and 5% FA and cured under ambient and accelerated carbonation. For mixes under accelerated carbonation, only concrete containing 8% MgO and 2% heavy hydrated magnesium carbonates obtained higher strength that that of the reference mix, containing 10% MgO. This was attributed to the lower water-demand and denser microstructure of heavy hydrated magnesium carbonates. Under natural curing conditions, reference concrete containing only 10% MgO obtained the maximum strength. The strength decreased with incorporation of hydrated magnesium carbonates, due to absence of sufficient CO_2_.
-	Unluer and Al-Tabbaa [54]	C	-	5	-	-	-	0.6–0.9	10	-	1–7	Under accelerated carbonation (10% CO_2_), MgO-concrete blocks with FA obtained strength values about 2 times higher than those of MgO mixes without FA. This was attributed to the higher porosity in FA mixes, leaving more space for hydrated magnesium carbonate formulation and strength development. Mixes with w/b 0.6 and 0.9 presented poor strengths due to low compaction and presence of saturated pores (preventing CO_2_ transportation and hydrated magnesium carbonate formulation), respectively. Maximum strength was obtained in MgO mixes with w/b 0.7, with and without FA. The strength of mixes gradually increased with increasing CO_2_ concentration from 0% to 20%. The strength development from 1 day to 7 days decreased with increasing CO_2_ concentration.
-	Gao et al. [46]	C	100	-	-	-	-	0.48	4–12	Calcination 1150 °C	3	Strength of concrete increased with increasing the autoclave time and decreased with increasing the autoclave temperature. Maximum strength was obtained in mixes with 4% MgO, followed by 8% and 12%.
-	-	-	70–100	30–50	-	-	-	0.48	4–12	Calcination 1150 °C	3	Strength of FA-concrete mixes decreased with increasing MgO content and increased with increasing autoclave time and temperature.
-	Gonçalves et al. [55]	M	100	-	-	-	-	0.50	0–20	Calcination 800 °C	28	Replacement of cement with 20% of MgO led to a decrease of the compressive strength (28%).
Flexural strength	Mo et al. [42]	M	100	-	-	-	-	0.5	5–8	Reactivity 50 s	3–90	Slight reduction of flexural strength with increasing the amount of MgO at all ages, when compared to that of reference mortars.
	-	-	25–100	20–35	20–40	-	-	0.5	5–8	Reactivity 50 s	3–90	At 3 days, the incorporation of slag and FA significantly decreased the strength of reference and MgO mixes. At 28 and 90 days, the strength of MgO-mortars with FA and slag became similar or even higher than that of reference or MgO mix without FA and slag.
-	Mo et al. [43]	M	100	-	-	-	-	0.5	8	Reactivity 50–400 s	3–90	The incorporation of four MgO types (reactivity values: 50, 100, 200, 400) had insignificant influence on the strength of mortars, regardless of MgO reactivity level and mortar curing age.
-	-	-	40–100	20	40	-	-	0.5	8	Reactivity 50–400 s	3–90	The strength of reference and MgO-mortars significantly decreased with the addition of FA and slag at 3 days. This difference highly decreased at 28 days. At 90 days, the strength of MgO-mortars with FA and slag was slightly higher than that of reference and MgO-mortars without FA and slag
-	Moradpour et al. [47]	M	100	-	-	-	-	0.5	1–5	-	7–90	The strength increased in mortars with nano-MgO, regardless of curing age and MgO content, when compared to that of conventional reference mortars. This trend was more obvious with lower MgO content.
-	Wei et al. [50]	M	-	-	-	10–40	-	0.5	60–90	-	3–28	Mortars were produced without OPC and with different amounts of microsilica and MgO. Maximum strength was obtained in mixes containing 30% microsilica and 70% MgO, at 3 and 28 days. This confirmed that full replacement of cement by microsilica and MgO can lead to production of mixes with the same strength as conventional mortars.
-	Mavroulidou et al. [45]	C	50–80	20–25	-	-	15	0.55	5–10	-	28	Strength of concrete mixes produced with metakaolin and different amounts of FA decreased or remained similar with increasing the amount of MgO.
-	Gao et al. [46]	C	100	-	-	-	-	0.48	4–12	Calcination 1150 °C	3	Strength of concrete increased with increasing the autoclave time and decreased with increasing the autoclave temperature. Maximum strength was obtained in mixes with 4% MgO, followed by 8% and 12%.
-	-	-	70–100	30–50	-	-	-	0.48	4–12	Calcination 1150 °C	3	Strength of FA-concrete mixes decreased with increasing MgO content and increased with increasing autoclave time and temperature.
-	Gonçalves et al. [55]	M	100	-	-	-	-	0.50	0–20	Calcination 800 °C	28	Flexural strength decreases (between 27% and 30%) can be observed with increasing replacement of cement with MgO, up to 20%.
Tensile strength	Mavroulidou et al. [45]	C	40–80	20–50	-	-	10-30	0.55	5-10	-	28	Strength of concrete mixes produced with different amounts of FA and metakaolin slightly decreased with increasing the amount of MgO.
Elastic modules	Choi et al. [44]	C	80	20	-	-	-	0.48–0.65	5	Calcination 1000 °C	28–360	Concrete mixes were produced with 20% FA, and different w/b (0.48, 0.65), and water cured for 28 and 360 days. The addition of MgO had almost no influence on the elastic modules of concrete mixes after 100, 200, and 300 cycles of freeze-thaw.
-	Gonçalves et al. [55]	M	100	-	-	-	-	0.50	0–20	Calcination 800 °C	28	There was a slight decrease (between 9% and 15%) in the mortars’ modulus of elasticity with 20% of MgO. This decrease is most likely due to the need to add more water to obtain equivalent workability levels.
Micro hardness	Mo and Panesar [56]	P	100	-	-	-	-	0.5	10–40	Calcination 800 °C	7–56	The microhardness of MgO-cement pastes was 25–52% higher than that of reference mixes, under carbonated condition. Exception occurred with cement paste having 40% MgO, tested at 7 days, obtaining 16% lower microhardness. Under non-carbonated condition, all MgO-cement pastes had similar or lower (6–36%) microhardness than that of reference mixes.

Legend: OPC = Ordinary Portland cement; S = Slag; FA = Fly ash; MS = Microsilica; MK = Metakaolin; w/b = Water to binder ratio; P = Paste; M = Mortar; C = Concrete.

**Table 4:** Durability of cementitious materials with MgO.

Test	Reference	Mix	Binder (%)				w/b	MgO		Age (days)	Results
			OPC	FA	S	MK		(%)	Properties		
Water permeability	Moradpour et al. [47]	M	100	-	-	-	0.5	1–5	-	28	The permeability decreased by 7–33% in mortars with nano-MgO, when compared to that of conventional reference mortars. This trend was more obvious in mortars with small MgO contents.
-	Dung and Unluer [51]	C	100	-	-	-	0.5–0.6	-	-	14–28	The incorporation of HCI (hydration agent-HA) and NaHMP (dispersion agent-DA) agents in concrete mixes containing MgO-based cement decreased water permeability by 2–42% when compared to that of the control mix. This trend was more pronounced in mixes containing HA and DA under ambient curing and in mixes with only DA under accelerated curing conditions.
-	Dung and Unluer [52]	C	100	-	-	-	0.6	-	-	14–28	The incorporation of different HA types (Magnesium acetate, magnesium chloride, hydrochloric acid) and HA concentrations (0.05–0.1 M) into MgO–concrete reduced the permeability by 2–74% when compared to that of conventional concrete mixes. This trend was more obvious under accelerated curing conditions than under ambient conditions.
Water absorption	Mavroulidou et al. [45]	C	45–80	20–25	-	15–25	0.55	5–10	Reactivity 976 s	28	Absorption of concrete mixes produced with different amounts of FA and metakaolin decreased with the addition of 5% MgO. The water absorption of 10% MgO–concrete mixes was higher than that of those with 5% MgO, but still lower than reference FA–metakaolin–concrete mixes. This was attributed to the lower compaction of mixes with higher MgO and metakaolin contents, caused by higher water demand.
Porosity	Mo and Panesar [56]	P	100	-	-	-	0.5	10–40	Calcination 800 °C	7–56	The total pore volume of cement pastes made with MgO was up to 32% lower and 10% higher than that of the reference paste, under carbonation and non-carbonation conditions, respectively.
-	Mo et al. [43]	P	10–60	20–40	-	-	0.4	20–60	Calcination 800 °C	28	Cement pastes were exposed to different CO_2_ periods and CO_2_ pressures (0.55 MPa for 3 and 15 h, and 0.10 MPa for 1 and 14 days). In mixes without CO_2_ exposure, the coarse pores and the total pore volume increased with increasing FA and MgO. Longer CO_2_ exposure and higher pressure decreased the porosity of all mixes. The CO_2_ exposure decreased fine pores (<0.2 μm) in mixes with higher FA and MgO content and coarser pores (0.2–2.0 μm) in mixes with higher cement content.
-	Liu et al. [39]	M	100	-	-	-	0.5	1–5	-	28	The total porosity of mortars with MgO was up to 19% higher than that of reference mix. Higher MgO content led to higher porosity in both 2-dimensionally restrained and unrestrained samples.
-	Mo et al. [42]	M	100	-	-	-	0.5	8	Reactivity 50 s	3–90	MgO-mortar had lower derivative porosity (size range 0.05–0.2 μm) and cumulative porosity than those of reference mix.
-	-	-	25–100	20–35	20–40	-	0.5	8	Reactivity 50 s	3–90	At 28 days, maximum porosity was obtained in reference mortar and mix with only MgO, at pore range of 0.02–0.2 μm. Taking into account pores smaller than 0.02 μm, MgO-mortars with 20–40% slag and 20–35% FA had higher porosity than that of reference mortar and mix with only MgO.
-	Pu and Unluer [48]	C	100	-	-	-	0.42–0.80	5–10	-	1–14	The porosity of concrete blocks made with 90–95% aggregates and 5–10% MgO or OPC was investigated under carbonation. At 1 day of age, maximum porosity was obtained in concrete with 10% MgO, followed by those with 5% MgO, 10% OPC, and both MgO (5%) and OPC (5%). At 14 days, maximum porosity decrements were obtained in concrete mixes containing 10% MgO or 10% OPC, when compared to those of the same mixes at 7 days.
-	Choi et al. [44]	C	80	20	-	-	0.48–0.65	5	Calcination 1000 °C	28–360	The addition of 5% MgO into concrete mixes produced with 20% FA and w/b of 0.65 led to higher total porosity at 28 days and lower at 360 days. This was attributed to the filler effect of MgO hydration products at longer ages. The addition of MgO decreased pores with 0.03–0.3 μm and 0.6–2.0 μm, and increased those with 0.01–0.03 μm and 0.3–0.6 μm, when compared to FA-concrete without MgO. MgO had similar influence in mixes with w/b of 0.48 and 0.65.
-	Unluer and Al-Tabbaa [53]	C	-	5	-	-	0.67–1.44	10	-	7	Cement was produced with 5–10% of MgO and 0–5% of heavy or light-hydrated magnesium carbonates. Concrete blocks were produced with 10% cement and 5% FA and cured under ambient and accelerated carbonation. Maximum porosity decreased after the carbonation exposure was obtained in concrete containing 8% MgO and 2% heavy hydrated magnesium carbonates, when compared to mixes with 5% MgO and 5% hydrated magnesium carbonates.
Carbonation	Mo et al. [41]	P	10–60	20–40	-	-	0.4	20–60	Calcination 800 °C	28	Cement pastes were exposed to different CO_2_ periods and CO_2_ pressures (0.55 MPa for 3 and 15 h, and 0.10 MPa for 1 and 14 days). Under 0.10 MPa CO_2_, cement pastes were partially or completely carbonated. Higher CO_2_ pressure increased the speed of carbonation. In addition, cement pastes with lower cement content (higher FA and MgO) showed higher carbonation.
-	Choi et al. [44]	C	80	20	-	-	0.48–0.65	5	Calcination 1000 °C	28–180	Concrete mixes were produced with 20% FA, and different w/b (0.48, 0.65), water cured for 28 and 360 days, and then carbonated for 28–180 days. Carbonation of concrete mixes water-cured for 28 days was not influenced by the MgO addition up to 180 carbonation days. The influence of MgO addition was more beneficial in mixes with 360 water curing days, at all carbonation ages. This was attributed to the MgO filler effect to decrease concrete porosity at longer ages.
-	Pu and Unluer [48]	C	100	-	-	-	0.42–0.80	10	-	1–14	The carbonation of concrete blocks made with 90–95% aggregates and 5–10% MgO or OPC was investigated at 1 and 14 days. For this, concrete samples were cut at three different levels (0, 20, 35 mm). In all cases, the carbonation was higher at longer ages (14 days) and on the outer side of the specimens. The carbonation degree of MgO–concrete samples (45%) was twice as high as the reference mix (22%) at 14 days.
-	Gonçalves et al. [55]	M	100	-	-	-	0.50	0–20	Calcination 800 °C	1–91	The carbonation depth increased with increasing content of MgO. The incorporation of 20% of MgO increases the carbonation depth between 139% and 483%, in the accelerated carbonation test for 28 days.
Chloride resistance	Choi et al. [44]	C	80	20	-	-	0.48–0.65	5	Calcination 1000 °C	28–360	Concrete mixes were produced with 20% FA, and different w/b (0.48, 0.65), and water cured for 28 and 360 days. The chloride ion migration coefficient increased with incorporating MgO in mixes water-cured for 28 days and decreased in those water-cured for 360 days. This was confirmed by porosity test, showing reduced total porosity and pores with 0.03–0.30 μm with the addition of MgO in mixes cured for 360 days.
Freezing and thawing	Choi et al. [44]	C	80	20	-	-	0.48–0.65	5	Calcination 1000 °C	28–360	Concrete mixes were produced with 20% FA, and different w/b (0.48, 0.65), and water cured for 28 and 360 days. The MgO might have had little effect on the freeze/thaw resistance of concrete. The authors justify that with the Mercury Intrusion Porosimetry (MIP) analysis, which indicates an absence of alteration of pores larger than 10 µm within MgO concrete.
Expansion	Liu et al. [39]	P	100	-	-	-	0.265	1–6	-	1–210	Cement pastes obtained higher expansion values with increasing the MgO content, when compared to that of reference mixes, at all curing temperatures (20 °C and 50 °C).
-	Mo et al. [37]	P	100	-	-	-	0.3	8	Calcination 900–1300 °C	1–300	Cement pastes were produced with five types of MgO, having different neutralization times (46–1966 s), calcination temperatures (900–1300 °C), and residence times (30–90 min), and cured at 20 °C and 40 °C in water. At 20 °C and age of 300 days, the expansion values obtained in mixes containing MgO of lower neutralization time (46–325 s) were significantly higher than those of reference paste and mixes with higher neutralization time MgO (1266–1966 s). At 40 °C, higher values were obtained in mixes containing MgO with higher neutralization times, when compared to that of reference mix.
-	Mo et al. [57]	P	100	-	-	-	0.28	2.75–7.00	Calcination 1000 °C	1–91	Cement pastes were produced with three blended expansive agents of dolomite and magnesite (1:0, 9:11, 3:7), and cured at 20 °C and 40 °C in water. At 20 °C, higher expansion values were obtained in mixes containing higher blended expansive agent amounts and magnesite ratios, when compared to those of reference pastes. Similar trend was observed at 40 °C. The expansion of cement pastes containing blended expansive agent at 40 °C were greater and faster than those of the same samples tested at 40 °C.
-	Mo et al. [43]	P	100	-	-	-	0.38	8	Reactivity 50–400 s	1–240	Cement pastes were produced with the incorporation of four MgO types (reactivity values: 50, 100, 200, 400 s), and cured at 20 °C and 38 °C in water. At 20 °C, MgO with higher reactivity led to higher expansion, when compared to that of the reference mix. By contrast, at 38 °C, mixes with lower reactivity MgO presented higher expansion.
-	-	-	40-100	20	40	-	0.5	8	Reactivity 50–400 s	1–240	For samples cured in both 20 °C and 38 °C water, the incorporation of FA and slag in MgO-pastes led to lower expansion values, due to the reduced MgO amount that was added by cement weight. Minimum expansion value was obtained by reference cement paste, followed by the one with FA, slag and the most reactive MgO (50 s).
-	Mo et al. [42]	P	100	-	-	-	0.38	5–8	Reactivity 50 s	1–210	Compared to reference mix, increasing the amount of MgO significantly increased the cement paste expansion, regardless of testing age.
-	-	-	25–100	20–35	20–40	-	0.38	5–8	Reactivity 50 s	1–210	Maximum expansion was observed in cement paste with 8% MgO, followed by the one containing 5% MgO, compared to that of the reference mix. Increasing the amount of slag and FA decreased the cement paste expansion. This was attributed to the MgO content reduction (which was added by weight of cement) with reduction of cement weight in FA and slag mixes.
-	Moradpour et al. [47]	P	100	-	-	-	0.25	1–5	-	1	The autoclave expansion of cement pastes produced with 1–5% nano-MgO was 0–33% higher than that of reference mortar, after being under autoclaved pressure for 3 h. Higher MgO amounts led to higher expansion.
-	Gao et al. [58]	C	100	-	-	-	0.48	4–12	-	2–180	Under the same curing conditions (90% RH, 20 °C), concrete mixes produced with higher MgO content registered significantly greater expansion, when compared to that of reference concrete.
-	Gao et al. [46]	C	100	-	-	-	0.48	4–12	Calcination 1150 °C	3	Expansion of concrete increased with increasing the amounts of MgO and temperature and time of autoclave.
-	-	-	70–100	30–50	-	-	0.48	4–12	Calcination 1150 °C	3	Under autoclave curing, expansion decreased with increasing FA content, regardless of MgO incorporation.
-	Gao et al. [58]	C	100	-	-	-	0.49–0.67	6–10	-	1–360	Concrete mixes were produced with purified MgO (conventional mix w/c 0.67) and calcined MgO (hydraulic mix w/c 0.49). Reference concrete in both conventional and hydraulic mixes shrunk up to 360 days. Expansion of conventional mixes was higher than that of hydraulic mixes, regardless of MgO content. Expansion increased with increasing MgO content, at all ages.
-	-	-	50–100	30–50	-	-	0.49–0.67	6–10	-	1–360	Expansion of conventional concrete mixes with 10% purified MgO and hydraulic mixes with 6% calcined MgO decreased with increasing FA content. This behaviour was attributed to FA reaction with Ca(OH)_2_ and Mg(OH)_2_ and reduced the expansion stress formulated by MgO.
Shrinkage	Mo et al. [43]	P	100	-	-	-	0.38	8	Reactivity 50–400 s	1–5	Cement pastes were produced with the incorporation of four MgO types (reactivity values: 50, 100, 200, 400 s). The autogenous shrinkage of cement pastes significantly reduced with decreasing the reactivity of MgO, when compared to that of reference mix. This was attributed to MgO hydration compensating the shrinkage.
-	-	-	40-100	20	40	-	0.5	8	Reactivity 50–400 s	1–5	The incorporation of slag and FA decreased the shrinkage of cement pastes. Mixes with FA, slag and MgO with reactivity value of 50 s slightly expanded at the end of the test, due to MgO hydration. The deformation curves in FA-slag-mortars with 50, 100, 200 and 400 MgO reactivity values needed 9, 22, 34, and 34 h to rise up after their steep fall.
-	Mo et al. [42]	P	100	-	-	-	0.38	5–8	Reactivity 50 s	1–5	Maximum autogenous shrinkage was obtained in the reference cement paste, followed by mixes containing 5% MgO, and 8% MgO. This was attributed to the compensation of the shrinkage by MgO hydration.
-	-	-	25–100	20–35	20-40	-	0.38	5–8	Reactivity 50 s	1–5	The autogenous shrinkage of mixes containing slag and FA were lower than that of the reference mix, regardless of the MgO addition. Mixes with 8% MgO and different amounts of FA and slag obtained no shrinkage by the end of the test. In fact, 8% MgO mixes with 20–40% slag and 20% FA exhibited small expansions after 24 h.
-	Jin et al. [49]	P	-	-	100	-	0.31	2.5–7.5	Reactivity 10–100 s	1–90	Compared to the shrinkage of alkali-activated slag cement at 90 days, the maximum decreases (26%) was obtained in mixes containing 7.5% high reactivity MgO, followed by those with 7.5% medium reactivity MgO, and 5.0% high reactivity MgO.
-	Gonçalves et al. [55]	M	100	-	-	-	0.50	0–20	Calcination 800 °C	1–91	The use of 15% of MgO as cement replacement led to notable decreases in shrinkage, up to less 400 μm/m in 91-day shrinkage measurements when compared to those of OPC concrete (439 μm/m at 91 days).
-	Kabir and Hooton [59]	C	100	-	-	-	0.50	0–15	Reactivity 55–210 s	1–180	7-day water curing of concrete prisms having different levels of MgO with reactivity of 55 s admixtures led to reductions in drying shrinkage: a reduction of more than 50% when 15% of MgO was used was observed. However, 210 s MgO did not mitigate shrinkage even at a 15% replacement level. Prisms cured in wet conditions for a month and having 15% of 55 s MgO ended up with a positive permanent expansion after 6 months of exposure to drying.

Legend: OPC = Ordinary Portland cement; S = Slag; FA = Fly ash; MS = Microsilica; MK = Metakaolin; w/b = water to binder ratio; P = Paste; M = Mortar; C = Concrete.

**Table 5:** Microscopic analysis results of cementitious materials with MgO.

Reference	Mix	Age (day)	Results
Mo et al. [37]	P	270	Cement pastes were produced with three types of MgO, having different neutralization times (46–1966 s). Hydration products in Mg(OH)_2_ were smaller and more irregular than those hydrated in water. Cracks were observed at the MgO particle interface.
Mo et al. [43]	P	90	Cement pastes were produced with the incorporation of two MgO types (reactivity values: 50, 400), FA and slag, and cured in 38 °C water. In mixes without FA and slag, MgO particles were surrounded by hydration products of cement. Sheets with similar features to brucite were observed, having some empty inner pores. In mixes with FA and slag, rims of pozzolanic reaction were mainly formulated around coarser slag particles. The finer slag particles appeared to be fully hydrated.
Mo et al. [41]	P	28	Cement pastes were produced with 20–40% FA and 20–60% MgO, and exposed to different CO_2_ periods and CO_2_ pressures (0.55 MPa for 3 h, and 0.10 MPa for 14 days). Exposing cement pastes to CO_2_ formulated interconnected rounded-shape products (Ca*_x_*Mg_1-*x*_CO_3_), bounding FA particles. Increasing CO_2_ pressure further densified the microstructure of cement pastes. Mixes with low cement content (10%) presented loose microstructure, which were enhanced with the CO_2_ exposure by interconnecting hydration products.
Abdalqader and Al-Tabbaa [40]	P	28	The microscopic analysis of mixes produced with OPC (25%, 75%), FA (15–25%), slag (45–60%), and MgO (5%, 10%) appeared to be similar. The formation of brucite was not detected, which may be consumed by slag. The characteristic platelets of hydrotalcite were also not defined due to its tiny size.
Jin et al. [49]	P	1–28	At 1 day, in the microstructure of alkali-activated slag cement, and those containing medium reactivity MgO and 2.5% high reactivity MgO, slag particles covered with reticulated C–S–H was observed. Dense C–S–H gels were found in mixes with 5.0% and 7.5% of high reactivity MgO, which means denser matrix microstructure, accelerated slag hydration, and higher strength. At 14 days, all mixes presented similar microstructures. An exception was detected in mixes with 2.5% high and low reactivity MgO, by showing fibrous Ht and hydrogarnet, respectively.
Moradpour et al. [47]	M	28	Mortars produced with 1%, 3% and 5% nano-MgO had denser microstructure than that of reference mix. This was attributed to the expansion and filler effects of MgO. No obvious differences were detected between mixes containing different MgO amounts. The backscattered electron mode of scanning electron microscopy (SEM) presented modified C–S–H in mixes with MgO.
Mo et al. [42]	M	28–90	The microscopy of a mortar produced with 5% MgO, 40% slag, and 20% FA showed that FA particles and their surroundings were covered with hydration products. The densified structure may have been useful for strength increase. Pozzolanic or hydraulic products were grown from outside to the unhydrated inside of slag particles. The densified slag–FA interface contributed to strength increase. Unlike finer slag particles, the larger ones were not fully hydrated.
Dung and Unluer [51]	C	14	MgO–concrete mixes were produced with and without HCI (hydration agent-HA) and NaHMP (dispersion agent-DA), and tested under ambient and accelerated carbonation condition. For ambient conditions, hydrated magnesium carbonate formation was 5–10 times higher in mixes containing HA and/or DA, when compared to that of concrete without additional agents, which eventually led to lower water absorption and higher compressive strength. Under accelerated carbonation, mixes containing HA presented a large amount of nesquehonite formation, as opposed to the domination of non-carbonated brucite under ambient condition.
Unluer and Al-Tabbaa [53]	C	7	Cement was produced with 5–10% of MgO and 0–5% of heavy or light hydrated magnesium carbonates. Concrete blocks were produced with 10% cement and 5% FA and cured under natural and accelerated carbonation. Under accelerated carbonation, reference mix produced with 10% MgO presented dypingite/hydromagnesite abundance, while other containing MgO and hydrated magnesium carbonates also showed nesquehonite. In mixes with 5% MgO and 2% heavy hydrated magnesium carbonates, dypingite/hydromagnesite and nesquehonite were observed to have increased, respectively, which eventually increased the mechanical strength of mixes containing 2%. In some mixes with 5% MgO and 5% light hydrated magnesium carbonates, uncarbonated brucite was defined, leading to lower concrete strength. Under natural curing, small quantities of brucite and uncarbonated MgO were detected, explaining the low strength of concrete mixes without carbonation acceleration.
Gao et al. [58]	C	28–360	Microscopic analysis of concrete mixes produced with 6% purified MgO showed that samples with 360 days obtained denser microstructures when compared to those of 28-day samples. This was attributed to MgO hydration products filling the pores at later ages.

Legend: FA = Fly ash; P = Paste; M = Mortar; C = Concrete.
